# Effect of NF-κB inhibitors on the chemotherapy-induced apoptosis of the colon cancer cell line HT-29

**DOI:** 10.3892/etm.2012.647

**Published:** 2012-07-24

**Authors:** TING LIU, DAN LIU, JING LIU, JI-TAO SONG, SHAN-LING GAO, HUI LI, LI-HONG HU, BING-RONG LIU

**Affiliations:** Department of Gastroenterology, The Second Affiliated Hospital of Harbin Medical University, Harbin, Heilongjiang 150086, P.R. China

**Keywords:** colon cancer, NF-κB inhibitor, chemotherapy, bortezomib, pyrrolidine dithiocarbamate, SN50

## Abstract

This study aimed to investigate the impact of the combined use of the nuclear factor-κB (NF-κB) inhibitors pyrrolidine dithiocarbamate (PDTC), bortezomib or SN50, and the chemotherapy agents arsenic acid (As_2_O_3_), fluorouracil (5FU), oxaliplatin or paclitaxel on the growth and apoptosis of HT-29 cells. Cell morphology was observed using inverted microscopy, and cell viability and apoptosis were assessed using the MTT assay and flow cytometry, respectively. The activities of NF-κB were analyzed by western blotting and electrophoretic mobility shift assay (EMSA). Cell growth was significantly inhibited by As_2_O_3_, oxaliplatin and paclitaxel in a time- and concentration-dependent manner (P<0.05), while 5FU inhibited cell growth in a time-dependent manner only (P<0.05). The growth inhibition rate and apoptosis induction ratio were increased following the combined treatment of the chemotherapy agent and NF-κB inhibitor. The expression of NF-κB p65 was upregulated when cells were treated with a chemotherapy drug, however it was downregulated following combined treatment or treatment with an NF-κB inhibitor alone. In conclusion, an NF-κB inhibitor combined with a chemotherapy drug effectively inhibited cell proliferation, induced cell apoptosis and inhibited NF-κB activity to enhance the chemotherapeutic sensitivity of HT-29 cells.

## Introduction

Nuclear factor-κB (NF-κB) was first identified as a regulator of the expression of the κ light-chain gene in murine B lymphocytes in 1986 (1), and a number of groups are currently researching the mechanism and effect of NF-κB. As an inducible nuclear transcription factor, NF-κB plays a key role in physiological or pathological conditions, and NF-κB and various signaling pathways regulate the expression of many genes involved in growth, differentiation, embryonic development, innate and adaptive immune responses, inflammation and apoptosis (2,3).

The occurrence and development of cancer is an extremely complex process which is affected by a variety of cytokines, signals and genetic changes. Cancer therapy has been ungoing for a long period of time, yet the effect of anticancer drugs remains inefficient. The primary cause may be chemotherapeutic resistance caused by either the deletion of a pro-apoptotic gene or the overexpression of an anti-apoptotic gene (4).

More recently, it has become clear that NF-κB signaling also plays a critical role in cancer development and progression. The activation of NF-κB results in the resistance of tumor cells to radiochemotherapy-induced cytotoxicity (3,5,6). NF-κB may also regulate tumor angiogenesis and invasiveness, and the signaling pathways that mediate its activation provide candidate targets for new chemopreventive and chemotherapeutic approaches (7,8). In the present study, we used the colon cancer cell line HT-29 to observe the effect of the NF-κB signaling pathway on apoptosis induced by chemotherapy drugs.

## Materials and methods

### Materials

The colon cancer cell line HT-29 was purchased from the Chinese Academy of Sciences (Shanghai, China). The arsenic acid sodium (As_2_O_3_) injection was purchased from Harbin Yida Pharmaceutical (Haerbin, Heilongjiang, China), fluorouracil (5FU) injection from the Tianjin Kingyork Group (Hedong, Tianjin, China), paclitaxel injection from Beijing Shiqiao Biological Pharmaceutical (Beijing, China) and oxaliplatin injection from Jiangsu Hengrui Medicine (Lianyungang, Jiangsu, China). The NF-κB inhibitors, bortezomib from Xian-Janssen Pharmaceutical (Beijing, China), SN50 from Alexis Biochemicals (San Diego, CA, USA) and ammonium pyrrolidine dithiocarbamate (PDTC) from Sigma (St. Louis, MO, USA) were used in this study. DMEM (high glucose) and fetal bovine serum were from HyClone (Logan, UT, USA), the Annexin V-PI apoptosis detection kit was from BD Biosciences, the SDA-PAGE gel configuration kit, RIPA cell lysates (strong), PMSF and BCA protein concentration determination kit were purchased from Beyotime Institute of Biotechnology (Haimen, Jiangsu, China). Protein liquid sample buffer 4X was purchased from Beijing Solarbio Science and Technology (Beijing, China). Prestained protein ladder was from Fermentas. The mouse monoclonal antibody to the NF-κB p65 subunit was obtained from Santa Cruz Biotechnology Inc. (Santa Cruz, CA, USA). Rabbit monoclonal to IKBα and rabbit polyclonal to survivin was from Abcam (Cambridge, MA, USA). Nuclear protein extraction kit and electrophoretic mobility shift assay (EMSA) kit were from Viagene Biotech (Los Angeles, CA, USA).

### Cell culture

Colon cancer HT-29 cells were grown in DMEM supplemented with 10% fetal bovine serum and 0.1% penicillin/streptomycin and maintained at 37°C in an atmosphere of 5% CO_2_. Cells were passaged to the next generation every two to three days and digested by 0.25% trypsin. Logarithmic growing cells were prepared.

### Cell viability assay

The cells were dispersed and plated at 6×10^4^ cells/well in 96-well microplates to determine the concentration and time-course of the response of HT-29 cells to As_2_O_3_, 5FU, oxaliplatin or paclitaxel, or combined with PDTC, bortezomib or SN50. Cell viability was assessed using an MTT assay following drug treatment at various concentrations or days in culture. The absorbance value (A) at 490 nm was read using a microplate reader (Thermo, Rockford, IL, USA). The inhibition rate was calculated as follows: Cell inhibition rate (%) = (1 - A of experiment well/A of positive control well) x 100%. Cell viability was assessed three times.

### Apoptosis assays and flow cytometry (FCM)

Following drug treatment for various hours, the HT-29 cell suspension was prepared using 0.125% trypsin and was rinsed and centrifuged with ice-cold PBS at 1,000 rpm for 5 min. The collected cells were treated with Annexin V-FITC or PI according to the manufacturer’s instructions (tube 1, unstained cells; tube 2, stained with PI; tube 3, stained with Annexin V-FITC; tube 4, stained with both Annexin V-FITC and PI) for 20 min away from the light, and Annexin fuorescence intensity was detected using FCM.

### NF-κB assays and western blotting

HT-29 cells were treated with a chemotherapy drug (5 mg/l As_2_O_3_, 300 mg/l 5FU, 20 mg/l oxaliplatin or 2.5 mg/l paclitaxel alone for 0, 3, 6, 12, 24 and 48 h, NF-κB inhibitor (50 μmol/l PDTC, 100 nmol/l bortezomib, 12.5 mg/l SN50) alone for 24 h, or combined with a chemotherapy drug for 24 h. The cell lysates were then prepared using standard methods. The protein concentration of each sample was measured using a BCA kit. Proteins from each sample were subjected to electrophoresis by SDS-PAGE, transferred onto polyvinylidene difluoride membranes (PVDF) with a transfer system (Bio-Rad, Hercules, CA, USA), and then blocked with a buffer containing 5% skimmed milk and 0.1% Tween-20 in Tris-buffered saline (TBST) at room temperature for 1 h. All antibodies were diluted in TBST. The membranes were incubated overnight with a primary antibody at 4°C, washed with TBST (3x10 min), followed by incubation with horseradish peroxidase (HRP)-conjugated secondary antibody for 1 h at room temperature, and then washed (3×10 min). Detection of chemiluminescence was performed with a DAB kit.

### EMSA

Nuclear proteins were extracted with a nuclear protein extraction kit in accordance with the manufacturer’s instructions. The protein concentration was determined using a BCA kit. The NF-κB probe was 5′-AGTTGAGGGGACTTTCCC AGGC-3′. Binding reactions were performed according to the non-radioactive EMSA kit. The specificity of the DNA and protein complex was confirmed by cold competition with a 50-fold excess of unlabeled NF-κB oligonucleotides. Binding reaction, gel electrophoresis, membrane transfer and immobilization, DNA binding, chemiluminescent reaction and imaging were performed sequentially.

### Statistical analysis

All data are expressed as means ± SD. Statistical analyses were performed using the Student’s t-test or ANOVA by SPSS 11.0. P<0.05 was considered to indicate a statistically significant result.

## Results

### Cell morphology

**Figure 1:**
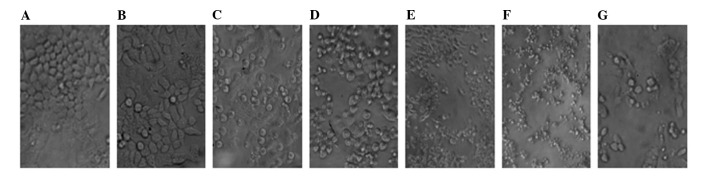
Cell morphologic changes. HT-29 cells in (A) normal growth or (B-E) treated with As_2_O_3_ alone for 3, 6, 12 and 24 h. The membranes successively became blurred, cells were shrunken and rounded to malapposition and even death. (F) Cells treated with As_2_O_3_ combined with PDTC (an NF-κB inhibitor) for 24 h were observe to be almost floating in the medium. (G) Cell treated with a chemotherapy drug for 48 h; only a few cells were alive. NF-κB, nuclear factor-κB; PDTC, pyrrolidine dithiocarbamate.

Cell morphologic changes were clearly observed using an inverted microscope. With an increase in time or increase in concentration of the chemotherapy drug, the HT-29 cell membranes gradually became blurred, rounding was reduced; cells were shrunken and rounded to malapposition and even death, while changes were more marked when the chemotherapy drug was combined with an inhibitor (Fig. 1).

### Chemotherapy-induced growth inhibition and apoptosis in the HT-29 cells

**Figure 2:**
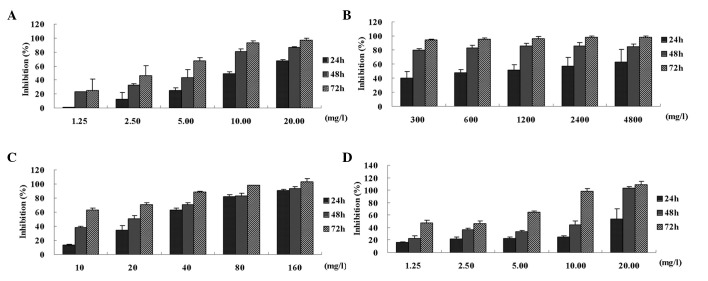
Effects of chemotherapy drugs on the viability of HT-29 cells. HT-29 cells were incubated with different concentrations of (A) As_2_O_3_, (B) 5FU, (C) oxaliplatin or (D) paclitaxel. When incubated for 24, 48 or 72 h, cell viability was quantifed by MTT. As_2_O_3_, oxaliplatin and paclitaxel inhibited cell proliferation in a time- and concentration-dependent manner (P<0.05), while 5FU only inhibited cell proliferation in a time-dependent manner (P<0.05). 5FU, fluorouracil.

**Figure 3:**
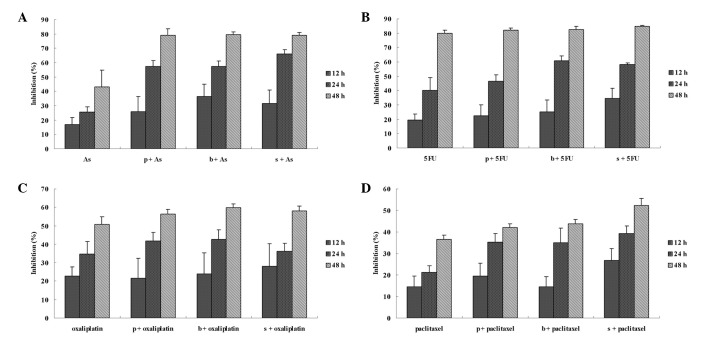
Effects of NF-κB inhibitors on chemotherapy-mediated growth inhibition. We selected 50 μmol/l PDTC (p), 100 nmol/l bortezomib (b) and 12.5 mg/l SN50 (s) as NF-κB inhibitors, and 5 mg/l As_2_O_3_ (As), 300 mg/l 5FU, 20 mg/l oxaliplatin and 2.5 mg/l paclitaxel as chemotherapy drugs. Incubation was carried out for 12, 24 and 48 h. The inhibition rate of the HT-29 cells was higher when the chemotherapy drugs were combined with an NF-κB inhibitor as compared with treatment with a chemotherapy drug alone. P>0.05 by ANOVA. NF-κB, nuclear factor-κB; PDTC, pyrrolidine dithiocarbamate; 5FU, fluorouracil.

**Figure 4:**
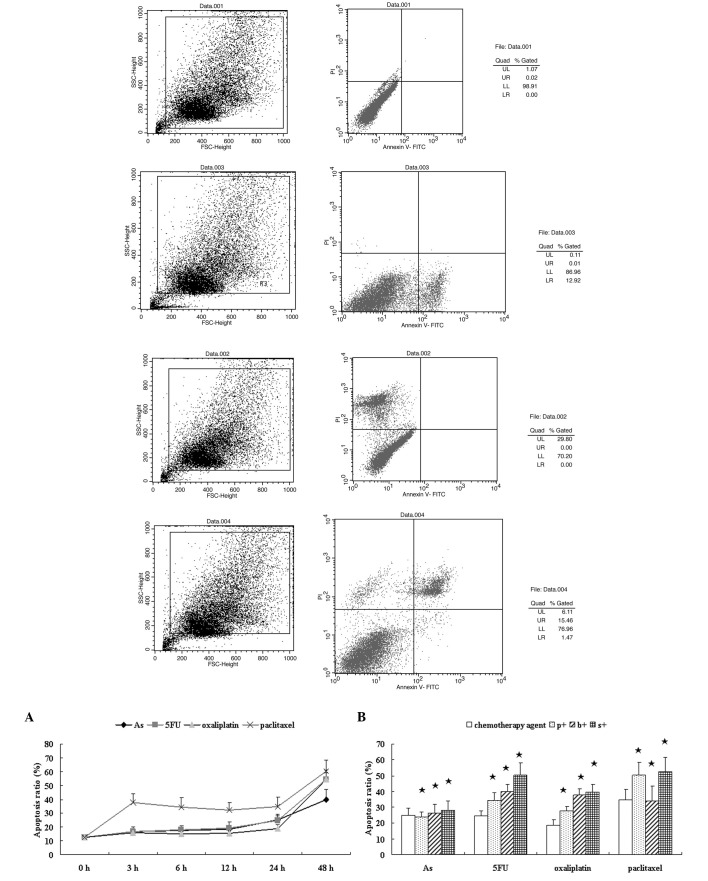
Effects of a chemotherapy drug alone or in combination with an NF-κB inhibitor on the apoptosis of HT-29 cells. (A) HT-29 cells were incubated with 5 mg/l As_2_O_3_, 300 mg/l 5FU, 20 mg/l oxaliplatin, 2.5 mg/l paclitaxel alone for 3, 6, 12, 24 and 48 h. (B) Cells were incubated with a chemotherapy drug alone for 24 h or combined with an NF-κB inhibitor: 50 μmol/l PDTC (p+), 100 nmol/l bortezomib (b+) or 12.5 mg/l SN50 (s+) for 24 h, and then Annexin fluorescence intensity was determined by FCM. As_2_O_3_, 5FU, oxaliplatin and paclitaxel induced cell apoptosis from the start of treatment. At 3, 6, 12 and 24 h the apoptosis rate continued; at 48 h the apoptosis rate increased suddenly, but late apoptosis was noted. NF-κB inhibitors enhanced chemotherapy-induced apoptosis when compared with the chemotherapy drug alone at 24 h; ^*^P>0.05. NF-κB, nuclear factor-κB; As, As_2_O_3_; 5FU, fluorouracil; PDTC, pyrrolidine dithiocarbamate; p+, chemotherapy drug combined with PDTC; b+, chemotherapy drug combined with bortezomib; s+, chemotherapy drug combined with SN50; FCM, flow cytometry.

Chemotherapy drugs are able to inhibit cell proliferation and promote apoptosis and NF-κB inhibitors are capable of enhancing chemotherapy-induced growth inhibition and apoptosis of HT-29 cells. As_2_O_3_, oxaliplatin and paclitaxel inhibited cell proliferation in a time- and concentration-dependent manner (P<0.05), while 5FU inhibited cell proliferation in a time-dependent manner (P<0.05; Fig. 2). The chemotherapy drugs promoted apoptosis in a time-dependent manner. NF-κB inhibitors enhanced chemotherapy drug-mediated growth inhibition (Fig. 3) and apoptosis induction (Fig. 4).

### NF-κB activity

**Figure 5:**
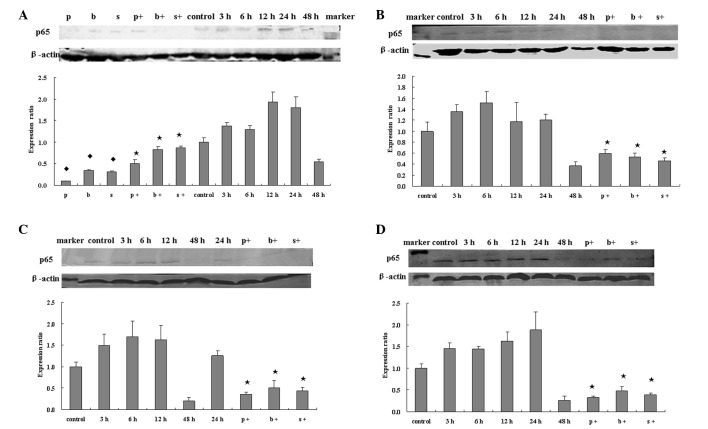
Expression of nuclear translocated activated p65 in western blotting. (A) Effect of As_2_O_3_ or NF-κB inhibitors alone for 24 h, and combined with each other. (B) Effect of 5FU alone or combined effect with NF-κB inhibitors. (C) Oxaliplatin and (D) 2.5 ml paclitaxel. It is obvious that p65 expression was upregulated when cells were treated with a chemotherapy drug while it was downregulated when an NF-κB inhibitor was used. ^•^P<0.05 compared with the control group. ^*^P<0.05 compared with the chemotherapy drug alone for 24 h group. NF-κB, nuclear factor-κB; p, 50 μmol/l PDTC; b, 100 nmol/l bortezomib; s, 12.5 mg/l SN50; p+, chemotherapy drug combined with PDTC; b+, chemotherapy drug combined with bortezomib; s+, chemotherapy drug combined with SN50; 5FU, fluorouracil.

**Figure 6:**
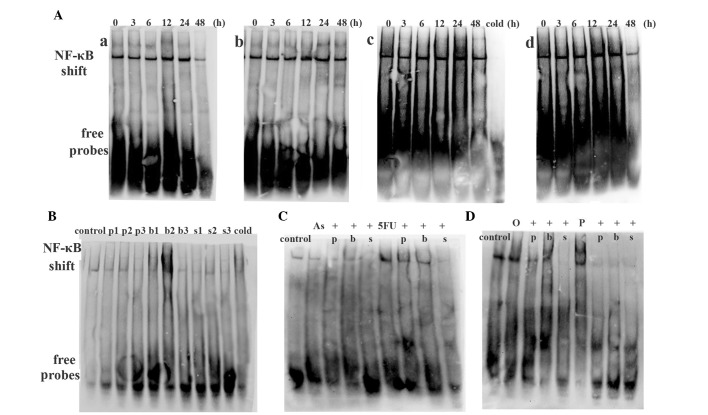
Expression of NF-κB in HT-29 cells as detected by EMSA. (A) Chemotherapy drug activated NF-κB activity: (a) 5 mg/l As_2_O_3_ alone for 3, 6, 12, 24 and 48 h; (b) 300 mg/l 5FU alone for 3, 6, 12, 24 and 48 h; (c) 20 mg/l oxaliplatin alone for 3, 6, 12, 24 and 48 h and a cold competition control; (d) 2.5 mg/l paclitaxel alone for 3, 6, 12, 24 and 48 h. (B) NF-κB inhibitors [50 μmol/l PDTC (p), 100 nmol/l bortezomib (b) or 12.5 mg/l SN50 (s)] decreased NF-κB activity. ‘1, 2, 3’ denote 6, 12 and 24 h respectively. (C) The effect of NF-κB inhibitors on As_2_O_3_- or 5FU-treated cells. (D) The effect of NF-κB inhibitors on oxaliplatin- or paclitaxel-treated cells. The untreated HT-29 cells or induced by a chemotherapy drug obtained p65 activity, and NF-κB inhibitors inhibited the NF-κB activity induced by the chemotherapy drug. As, As_2_O_3_; 5FU, fluorouracil; O, oxaliplatin; P, paclitaxel. EMSA, electrophoretic mobility shift assay; NF-κB, nuclear factor-κB.

NF-κB activity was activated by chemotherapy drugs and was reduced by NF-κB inhibitors. The expression of total NF-κB p65 was detected by western blotting and that of nuclear translocated activated p65 by EMSA (Figs. 5 and 6). Both levels increased following treatment with a chemotherapy drug for 3, 6, 12 and 24 h, but were weakened at 48 h. p65 expression was significantly inhibited compared with chemotherapy drug treatment alone.

## Discussion

Colorectal cancer is a common malignancy and is strongly associated with a Western lifestyle. In the past several decades, much has been learned about the dietary, lifestyle and medical risk factors for this malignancy (9,10). It is the third leading cause of cancer-related death in individuals of each gender and the second overall in men and women combined in the US (11). In China, with the improvement of living standards and changes in diet, the incidence of colorectal cancer has gradually increased, but half of colorectal cancer treatment fails. Even in regions with improved living conditions the overall 5-year survival rate is approximately 30%.

NF-κB is an important nuclear transcription factor, comprised of a complex system. It exists in a variety of cells, and plays a great role in the gene regulation of inflammation, immune response, cell proliferation and apoptosis (12). In resting cells, NF-κB is sequestered in the cytoplasm in association with inhibitory proteins IκB which cover the nuclear localization signal (NLS) of NF-κB. When cells are subjected to a variety of stimuli, including bacteria or virus infection, inflammatory cytokines, TNF, LPS, ultraviolet ray and ionizing radiation, IκB is phosphorylated, ubiquitinated and is quickly degraded by proteasomes, NF-κB is released and activated, then translocates into the nucleus and binds to the promoter region of target genes to regulate a series of gene expression patterns involved in the control of different cellular responses (13,14).

The anti-apoptotic ability of NF-κB was identified by chance. Beg *et al* (15) studied the function of NF-κB using gene knockout mice, RelA was knocked out from embryonic stem cells to study the influence on mouse survival, and the embryos died on the 15th or 16th day; autopsies and pathological inspection revealed a large quantity of liver cell apoptosis. The identification of NF-κB involvement in cell apoptosis has aroused great interest. Numerous groups are currently researching NF-κB and have found that NF-κB plays a key role in cancer anti-apoptotic mechanisms (3,16,17); Wu *et al* (18) found that adenosine arrested hepatocellular carcinoma cells in the G0–G1 phase of the cell cycle, enhanced the activity of caspase-3 and upregulated p53, but at the same time upregulated NF-κB p65 expression and downregulated Bcl-2 expression. NF-κB inhibition of PDTC decreased p65 expression, enhanced cell apoptosis ratio and increased caspase-3 activity. NF-κB may play an anti-apoptotic role in adenosine-induced HepG2 cytotoxicity; Furuta *et al* (19) applied NBD peptide which disrupted the association of NF-κB essential modulator (NEMO) with IκB kinases on oral squamous cell carcinoma, and the conclusion was that NBD peptide treatment inhibited TNFα-induced, and constitutive, NF-κB activation, increased apoptosis and suppressed proliferation. Zhu *et al* (20) investigated the antitumor effects of the NF-κB inhibitor SN50 in gastric carcinoma SGC-7901 cells and revealed that NF-κB inhibition triggers an impairment of cell proliferation and the induction of apoptosis of cancer cells. Blocking NF-κB may increase the expression of p53 and induce pro-apoptotic and autophagic proteins.

Many different sites may be exploited to block NF-κB activation in the NF-κB pathway. PDTC is a type of metal chelating agent and antioxidant. It inhibits the release of the IκB subunit from the cytoplasm and prevents the separation between IκB and NF-κB to inhibit the activation of NF-κB (21). Proteasome inhibitor bortezomib inhibits IκB degradation following phosphorylation and ubiquitination (22,23) and SN50 inhibits coupling between NF-κB and the effective DNA (24). The effect site of each of the inhibitors is closer, sequentially, to the terminal of the NF-κB pathway and the specificity increases accordingly.

Different chemotherapy drugs have their own mechanisms. The mechanism of As_2_O_3_ is unclear, but it induces apoptosis and inhibits telomerase activity to inhibit cell division; 5FU is classified as an antimetabolite which is a cell-cycle-specific chemotherapy drug and attacks cells at specific phases in the cycle. 5FU and its metabolites are similar to normal substances within the cell. When they are incorporated into cells, they inhibit essential biosynthetic processes, or are incorporated into the macromolecular DNA and RNA to inhibit their normal fuction. Oxaliplatin is an alkylating agent which is cell-cycle non-specific and is most active in the resting phase of the cell. It forms a coordination metal salt complex and inhibits DNA synthesis in cancer cells. Paclitaxel is a taxane plant alkaloid and an antimicrotubule agent which is cell-cycle specific and attacks cells during various phases of division. It stabilizes the microtubule structures and inhibits spindle formation, which are part of the cell division and replication apparatus, resulting in cell death.

In our study, we applied As_2_O_3_, 5FU, oxaliplatin, paclitaxel alone or combined with PDTC, bortezomib or SN50 to the colon cancer cell line HT-29. We confirmed that As_2_O_3_, oxaliplatin and paclitaxel inhibited cell proliferation in a time- and concentration-dependent manner, while 5FU only inhibited cell proliferation in a time-dependent manner (Fig. 2). NF-κB inhibitors had enhanced chemotherapy-mediated growth inhibition (Fig. 3). The cell apoptosis rate was also higher when the chemotherapy drug was combined with an NF-κB inhibitor. The inhibitors, 50 μmol/l PDTC, 100 nmol/l bortezomib and 12.5 mg/l SN50, suppressed the NF-κB expression of the tumor cells themselves, which was stimulated by chemotherapy (P<0.05). The result of NF-κB nuclear transfer tested by EMSA was consistent with the total protein expression tested by western blotting. Therefore, we come to the conclusion that the NF-κB inhibitors, PDTC, bortezomib and SN50, inhibit NF-κB activation and improve the cell inhibition rate and apoptosis ratio to influence the effect of chemotherapy on HT-29 cells. The NF-κB protein expression was inhibited by NF-κB inhibitors significantly compared with the chemotherapy drugs (P<0.05), while the cell inhibition rate and apoptosis ratio were improved (P>0.05).

When 5 mg/l As_2_O_3_, 300 mg/l 5FU, 20 mg/l oxaliplatin or 2.5 mg/l paclitaxel was applied to HT-29 cells alone, the total protein expression of NF-κB was increased, and the highest increase was 1.93±0.23, 1.51±0.21, 1.70±0.37 and 1.88±0.41 times, respectively. The inhibitors, 50 μmol/l PDTC, 100 nmol/l bortezomib, 12.5 mg/l SN50, suppressed the NF-κB expression which was stimulated by the tumor cells themselves; the degrees of inhibtion were 0.11±0.00, 0.35±0.01 and 0.31±0.03 times, respectively (P<0.05). NF-κB inhibitors were able to inhibit the NF-κB expression which was stimulated by chemotherapy (P<0.05). The result of NF-κB nuclear transfer tested by EMSA was consistent with the total protein expression tested by western blotting.

We conclude that the NF-κB inhibitors, PDTC, bortezomib and SN50, inhibit NF-κB activation, improve the cell inhibition rate and apoptosis ratio to influence the effect of chemotherapy on HT-29 cells. The NF-κB protein expression was inhibited by NF-κB inhibitors significantly compared with the chemotherapy drugs (P<0.05), while the cell inhibition rate and apoptosis ratio were improved (P>0.05). This differs from other research, maybe due to the effect time, dose or the two combined. Therefore, the best concentration and incubation time of the NF-κB inhibitor, and whether the effect would be improved when cells had acquired chemotherapy drug resistance, require further investigation.

## References

[1] Sen R, Baltimore D (1986). Multiple nuclear factors interact with the immunoglobulin enhancer sequences. Cell.

[2] Zandi E, Karin M (1999). Bridging the gap: composition, regulation, and physiological function of the IkappaB kinase complex. Mol Cell Biol.

[3] Baldwin AS (2001). Control of oncogenesis and cancer therapy resistance by the transcription factor NF-kappaB. J Clin Invest.

[4] Gottesman MM, Fojo T, Bates SE (2002). Multidrug resistance in cancer: role of ATP-dependent transporters. Nat Rev Cancer.

[5] Wang CY, Guttridge DC, Mayo MW, Baldwin AS (1999). NF-kappaB induces expression of the Bcl-2 homologue A1/Bfl-1 to preferentially suppress chemotherapy-induced apoptosis. Mol Cell Biol.

[6] Wang CY, Mayo MW, Korneluk RG, Goeddel DV, Baldwin AS (1998). NF-kappaB antiapoptosis: induction of TRAF1 and TRAF2 and c-IAP1 and c-IAP2 to suppress caspase-8 activation. Science.

[7] Karin M (2006). Nuclear factor-kappaB in cancer development and progression. Nature.

[8] Mitsiades N, Mitsiades CS, Poulaki V, Anderson KC, Treon SP (2002). Intracellular regulation of tumor necrosis factor-related apoptosis-inducing ligand-induced apoptosis in human multiple myeloma cells. Blood.

[9] Poynter JN, Haile RW, Siegmund KD, Campbell PT, Figueiredo JC, Limburg P, Young J, Le Marchand L, Potter JD, Cotterchio M, Casey G, Hopper JL, Jenkins MA, Thibodeau SN, Newcomb PA, Baron JA, Colon Cancer Family Registry (2009). Associations between smoking, alcohol consumption, and colorectal cancer, overall and by tumor microsatellite instability status. Cancer Epidemiol Biomarkers Prev.

[10] Bostick RM, Potter JD, Kushi LH, Sellers TA, Steinmetz KA, McKenzie DR, Gapstur SM, Folsom AR (1994). Sugar, meat and fat intake, and non-dietary risk factors for colon cancer incidence in Iowa women. Cancer Causes Control.

[11] Chan AT, Giovannucci EL (2010). Primary prevention of colorectal cancer. Gastroenterology.

[12] Kim HJ, Hawke N, Baldwin AS (2006). NF-kappaB and IKK as therapeutic targets in cancer. Cell Death Differ.

[13] Legrand-Poels S, Schoonbroodt S, Matroule JY, Piette J (1998). NF-kappaB: an important transcription factor in photobiology. Photochem Photobiol B.

[14] Romano MF, Lamberti A, Bisogni R, Tassone P, Pagnini D, Storti G, Del Vecchio L, Turco MC, Venuta S (2000). Enhancement of cytosine arabinoside induced apoptosis in human myeloblastic leukemia cells by NF kappaB/Rel-specific decoy oligodeoxy-nucleotides. Gene Ther.

[15] Beg AA, Sha WC, Bronson RT, Ghosh S, Baltimore D (1995). Embryonic lethality and liver degeneration in mice lacking the RelA component of NF-kappaB. Nature.

[16] Holmes-McNary M, Baldwin AS (2000). Chemopreventive properties of trans-resveratrol are associated with inhibition of activation of IkappaB kinase. Cancer Res.

[17] Yemelyanov A, Gasparian A, Lindholm P, Dang L, Pierce JW, Kisseljov F, Karseladze A, Budunova I (2006). Effects of IKK inhibitor PS1145 on NF-kappaB function, proliferation, apoptosis and invasion activity in prostate carcinoma cells. Oncogene.

[18] Wu LF, Li GP, Su JD, Pu ZJ, Feng JL, Ye YQ, Wei BL (2010). Involvement of NF-kappaB activation in the apoptosis induced by extracellular adenosine in human hepatocellular carcinoma HepG2 cells. Biochem Cell Biol.

[19] Furuta H, Osawa K, Shin M, Ishikawa A, Matsuo K, Khan M, Aoki K, Ohya K, Okamoto M, Tominaga K (2012). Selective inhibition of NF-κB suppresses bone invasion by oral squamous cell carcinoma in vivo. Int J Cancer.

[20] Zhu BS, Xing CG, Lin F, Fan XQ, Zhao K, Qin ZH (2011). Blocking NF-κB nuclear translocation leads to p53-related autophagy activation and cell apoptosis. World J Gastroenterol.

[21] Si X, McManus BM, Zhang JC, Yuan J, Cheung C, Esfandiarei M, Suarez A, Morgan A, Luo HL (2005). Pyrrolidine dithiocarbamate reduces coxsackievirus B3 replication through inhibition of the ubiquitin-proteasome pathway. J Virol.

[22] Caravita T, de Fabritiis P, Palumbo A, Amadori S, Boccadoro M (2006). Bortezomib: efficacy comparisons in solid tumors and hematologic malignancies. Nat Clin Pract Oncol.

[23] Nencioni A, Grünebach F, Patrone F, Ballestrero A, Brossart P (2007). Proteasome inhibitors: antitumor effects and beyond. Leukemia.

[24] Orange JS, May MJ (2008). Cell penetrating peptide inhibitors of nuclear factor-kappaB. Cell Mol Life Sci.

